# Fall Sickness

**Published:** 1874-10

**Authors:** 


### FALL SICKNESS.

It is a common observation that many persons get sick after returning home from their summer recreation, arising in part from living a less active life than when in the country, and yet eating with the appetite country air and country exercise give; the result being, that more power is taken into the stomach than the system requires; is imperfectly digested, making imperfect or bad blood; and as this blood goes to every part of the body to renew and invigorate, it fails to answer the purpose, because it is not a good material, does not fully come up to the needed requirements; this deranges the machinery of life, and innumerable "symptoms" manifest themselves. A good plan, therefore, would be to avoid eating between meals, to take nothing later than noon but a slice or two of bread and butter with a cup of tea, no dessert at dinner; no meat one day in the week, and nothing for breakfast every other morning but a cup of hot drink, cold bread and butter, and, as dessert, a generous plate of some kinds of ripe, fresh, perfect fruits or melons. The truth is, if all who live mostly in doors would adopt this plan of eating the year round, it would abate half the sickness.

Another cause of autumnal diseases to returning tourists is, that remaining too long in low, flat, or damp localities, the system becomes impregnated with malaria or miasmatic impurities, making the blood poor, bad, or impure otherwise, with the result, that slight indiscretions in eating, exercise or exposure, light up the fires of disease; and if there is not actual fever and ague, or the more disagreeable and intractable dumb ague, there is a general feeling of discomfort, of dire greediness, lasting well on into the winter. Little children are especially liable to obscure and undefined ailments; you cannot tell exactly what is the matter with them, but you know they are not well. These things can be pretty well avoided by taking two precautions. The signal for leaving the springs and seashore is the cool nights and mornings. As soon as persons get back home a thicker material of woolen flannel should be worn next the body, and heavier garments for the legs and feet, of children especially. In addition, there ought to be kindled a blazing fire on the hearth in the family room every morning and at sundown; this takes off the dampness and sharpness of the morning and evening air, and drives away those particles contained therein, which are the immediate cause of all ordinary autumnal diseases.

If persons in the country, where intermittents prevail, would adopt this precaution from the middle of September, and would take their breakfast before going out of doors in the morning, fever and ague would almost entirely disappear as a prevailing disease. Let any family try this for a single autumn, and be governed thereafter by the result. Many a tidy housekeeper brings sickness into the family for half the winter, with its suffering, its expense and its extra work, trouble and discomfort, by putting off the kindling of fires until the latest day possible, so as to escape the inconvenience of dust and ashes; and for this trifling reason she will have her family shiver half the morning. I knew an old lady of wealth who kept her husband warm by piling folded blankets in his lap. Let a brightly blazing fire be found in any room in the house of autumn mornings, and instinct will bring the little children there; it will be the most "popular" apartment in the whole establishment. No one can be cheery and conversible, if on the verge of a chill, nor even in a good humor. All fevers and all colds are preceded by a chill; yet, in the way above named, thousands are recklessly exposed to them, for the sake of avoiding a little trouble from the flying about of the dust and ashes incident to kindling fires.

A substitute might be found in lighting up the furnace, but that heats the whole house and keeps it extra heated the entire day, when it is only wanted for an hour or two in the early morning, and at sundown.

